# Detection of entanglement in asymmetric quantum networks and multipartite quantum steering

**DOI:** 10.1038/ncomms8941

**Published:** 2015-08-03

**Authors:** D. Cavalcanti, P. Skrzypczyk, G. H. Aguilar, R. V. Nery, P.H. Souto Ribeiro, S. P. Walborn

**Affiliations:** 1 ICFO-Institut de Ciencies Fotoniques, Mediterranean Technology Park, Avenue Carl Friedrich Gauss, 3, Castelldefels, 08860, Barcelona, Spain.; 2 H. H. Wills Physics Laboratory, Tyndall Avenue, University of Bristol, Bristol BS8 1TL, UK.; 3 Instituto de Fsica, Universidade Federal do Rio de Janeiro, CP 68528, 21941-972 Rio de Janeiro, Brazil.

## Abstract

The future of quantum communication relies on quantum networks composed by observers sharing multipartite quantum states. The certification of multipartite entanglement will be crucial to the usefulness of these networks. In many real situations it is natural to assume that some observers are more trusted than others in the sense that they have more knowledge of their measurement apparatuses. Here we propose a general method to certify all kinds of multipartite entanglement in this asymmetric scenario and experimentally demonstrate it in an optical experiment. Our results, which can be seen as a definition of genuine multipartite quantum steering, give a method to detect entanglement in a scenario in between the standard entanglement and fully device-independent scenarios, and provide a basis for semi-device-independent cryptographic applications in quantum networks.

## Abstract (web summary)

Quantum communications operate with shared multipartite entangled states, and this has to be certified in a setting where not all parties are trusted in the same way. Here the authors propose a method to certify multipartite entanglement in asymmetric scenarios and demonstrate it in an optical experiment.

## Graphical abstract

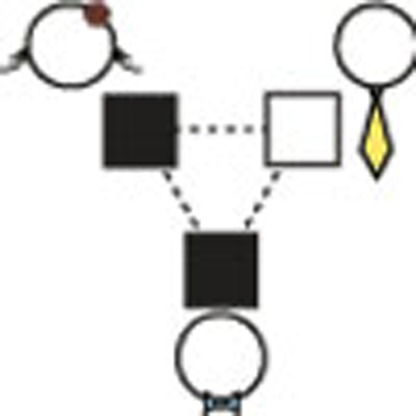

The most widely used techniques to detect entanglement rely either on having knowledge of the quantum state, obtained through quantum state tomography, or on the use of measurements that constitute an entanglement witness^1^. A frequently disregarded assumption behind these methods is that the measurements and devices used are well characterized. However, a mismatch between the theoretical description of the measurements and their actual implementation may lead to erroneous conclusions about the presence of entanglement^2^. A way of avoiding this assumption is to use device-independent techniques^3^, where the measuring devices are not trusted to behave as expected, and no specific description of the experimental observables is assumed. In this approach, the measurement devices are considered as black boxes that the parties can access with classical inputs (corresponding to the measurement choices) that provide classical outputs (considered as the measurement results). The presence of entanglement is then verified analysing the correlation statistics between the data lists corresponding to the measurement results. The violation of Bell inequalities^4^ certify the presence of entanglement in this scenario, which can be thought of as a device-independent entanglement witness. The device-independent approach is especially important in adversarial scenarios, such as device-independent quantum key distribution^5^, where an adversary can use a mismatch between the real implementation of the protocol and its description to fake its performance^6^,^7^,^8^. However, the violation of a Bell inequality requires a high degree of correlation between the parties tolerating then very low levels of noise and demanding highly efficient detectors and high-quality entangled states^3^.

An intermediate scenario between the standard and the device-independent cases is that of quantum steering^9^,^10^. This is the situation where, in the bipartite case, one of the parties uses a trusted measuring device but the other does not. As such, we refer to this approach as the semi-device-independent one. Apart from the fundamental importance of characterizing separability in different scenarios, quantum steering appears as a practical situation that is less demanding experimentally than the device-independent approach. It requires fewer assumptions than the standard case and lower strength for the quantum correlations to be witnessed or certified. For these reasons, the study of quantum steering, including its applications^11^,^12^ and experimental demonstrations^13^,^14^,^15^,^16^,^17^,^18^,^19^, has increased rapidly over recent years.

In the multipartite case, much knowledge has been acquired concerning standard entanglement detection^1^ and the device-independent case^20^,^21^,^22^,^23^,^24^,^25^,^26^,^27^. However, only few results were found in the semi-device-independent case. For instance, ref. 28 provides inequalities to rule out fully separable states, ref. 29 developed a probabilistic protocol to detect the presence of a particular multipartite entangled state, and ref. 30 discussed a hybrid model where each party is sometimes trusted and sometimes untrusted (see also refs 31, 32 for recent experimental demonstrations).

Apart from the fundamental problem of understanding multipartite quantum correlations, extending the semi-device-independent approach to the multipartite scenario is also relevant for practical purposes. As technology advances it will be possible to establish large quantum networks. These networks will be asymmetric in many cases, depending on the experimental capabilities of each station, the specific architecture of the implemented protocols and unavoidable limitations that the set-up may impose. Let us give a few examples. Consider, for instance, prepare-and-measure cryptographic protocols in which some parties hold the sources of quantum systems and some others act as the receivers who measure these systems. Since the senders do not receive any external signals, they may consider that no eavesdropper is manipulating their apparatuses. Thus, any error they observe (as, for example, due to detection inefficiencies) can be attributed to the apparatuses' imperfections. The receivers, on the other hand, are given systems that may have been intercepted by an eavesdropper, who may use extra degrees of freedom that are not considered by the receiver (see refs 6, 7, 8 for examples). In this case, the receivers' apparatuses cannot be considered trusted. Another scenario is that in which no reference frame can be established by some of the stations^33^. In this case, the measurement directions that some of the parties implement are not known, and may as well be considered as untrusted. Finally, quantum key distribution systems and quantum randomness generators are nowadays at the commercial level. Clearly, the general consumers of these products are not capable of reverse engineering the devices, and may not want to trust their providers.

Here we propose a general method to detect all kinds of entanglement that can be present in a quantum network, where some of the parties use untrusted measurements and must use data lists. We show how the different types of entanglement constrain the corresponding observed experimental data and present an efficient method to obtain semi-device-independent entanglement witnesses in the form of multipartite steering inequalities. We furthermore implement this method in a proof-of-principle optical experiment and demonstrate the violation of tripartite steering inequalities in both scenarios where either one or two parties perform untrusted measurements. Finally, we also quantify the advantage that the present approach provides over the device-independent one in terms of tolerance to noise.

## Results

### Semi-device-independent test of multipartite entanglement

**Figure 1:**
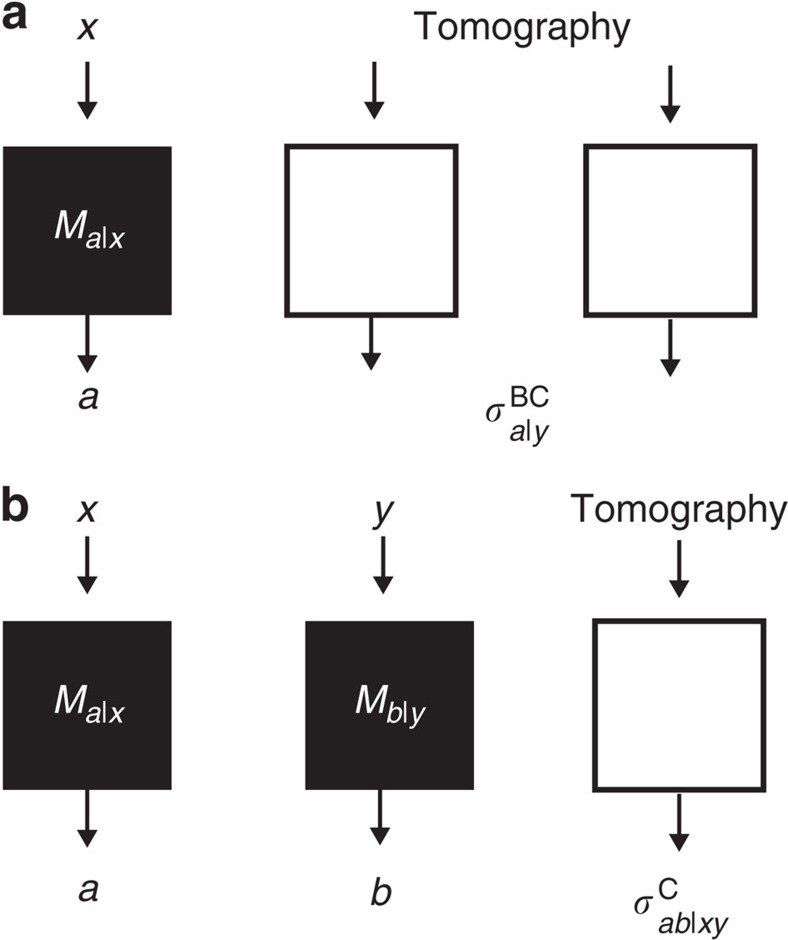
Asymmetric tripartite networks where untrusted devices are treated as black boxes with classical inputs and outputs. (**a**) One untrusted party scenario: Alice, who holds an untrusted device, treats it as a black box in which she inputs *x* (the measurement choice) and receives an output *a* (the measurement outcome). This procedure corresponds mathematically to applying some unknown measurement operator 
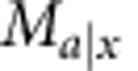
 to the shared tripartite quantum state, which produces a post-measurement state 
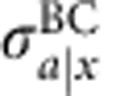
 at Bob and Charlie's locations. (**b**) A similar situation occurs in the two untrusted party scenario, when both Alice and Bob perform untrusted measurements (corresponding to unknown measurement operators 
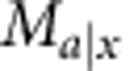
 and 
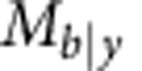
, respectively) preparing quantum states 
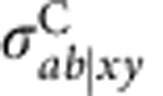
 on Charlie's system.

We start by explaining the scenario considered here, which consists of a quantum network on *N* parties sharing an unknown system in state *ρ* (see Fig. 1). Some of the parties perform measurements that are uncharacterised, or untrusted, while others have total control over their measurement apparatuses. Those parties who do not trust their apparatuses treat them as black boxes in which they can provide classical inputs (corresponding to the choice of measurement settings) and receive classical outputs (corresponding to the measurement outcomes). Notice that not even the Hilbert space dimension of these systems is assumed. The parties that trust their measurements can actually implement quantum state tomography, and reconstruct the density matrix they hold after the untrusted parties announce their measurement choices and outcomes. On the basis of this knowledge, the goal is to decide whether the original state *ρ* had some kind of entanglement.

In the general case of *N* parties there will be several semi-device-independent cases, depending on which parties are trusted. For simplicity, in what follows we will explain our method for the case of detecting genuine multipartite entanglement in a tripartite system. This case contains all the basic ingredients needed to understand both how to detect other types of entanglement and how to treat systems composed of more parties. These procedures are described in detail in the Supplementary Notes 1–3.

Let us consider that an unknown tripartite state *ρ*^ABC^ is distributed between three parties: Alice, Bob and Charlie. Two semi-device-independent cases arise: (i) when only one party's device is untrusted and (ii) when two parties' devices are untrusted. Let us consider the first case, supposing that Alice holds the untrusted device. In this case, there is no assumption on Alice's measurements and we describe them with some unknown measurement operators 
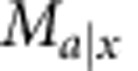
, where the subscript *x* labels the measurement choices and *a* the possible outcomes. Not even the dimension of Alice's subsystem is assumed. Since Bob and Charlie trust their apparatuses they can perform tomography and determine their (unnormalized) conditional states 
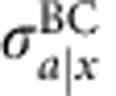
 as


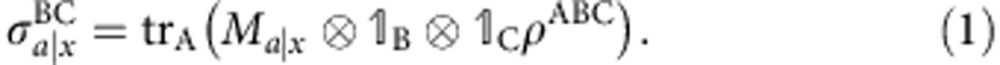


The set of unnormalized states 
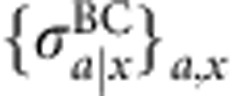
 is called an assemblage and contains all the information obtainable in this situation, as it encodes both the probability that Alice obtains the result *a* given that she made the measurement *x*, as 
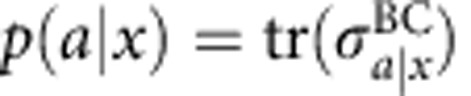
, as well as the corresponding conditional state *ρ*_*a*|*x*_^BC^=*σ*_*a*|*x*_^BC^/*p*(*a*|*x*).

The second situation is when two parties, say Alice and Bob, have untrusted devices. In this situation, Bob's measurement is also treated as a black box performing measurements associated with unknown measurement operators 
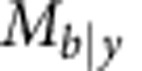
, while Charlie can tomographically determine the assemblage


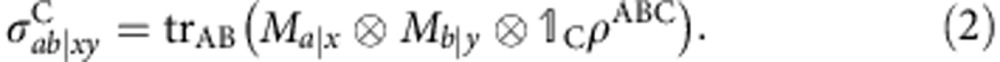


The probability distributions of Alice and Bob's measurements is encoded in 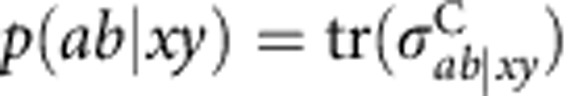.

If the initial state *ρ*^ABC^ contains no genuine multipartite entanglement, that is, it is biseparable, then it has the form


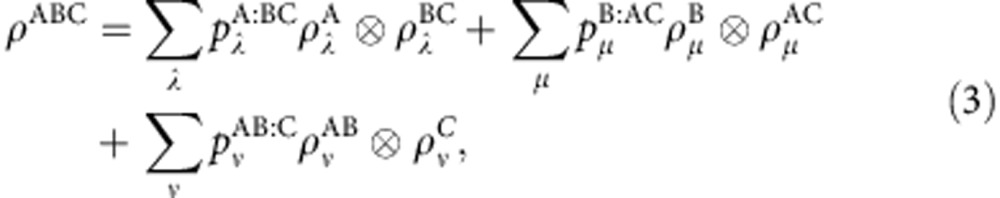


where 
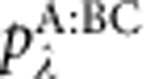
, 
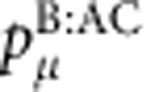
 and 
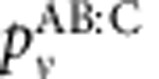
 are probability distributions. Then, the assemblages (1) and (2) have the form


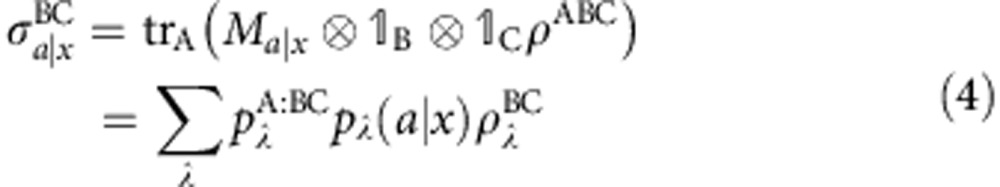



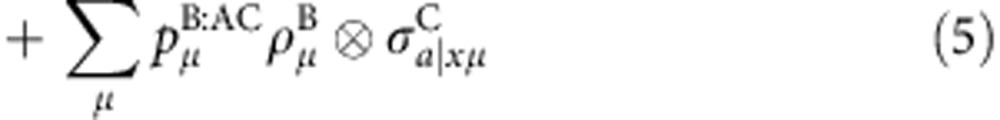



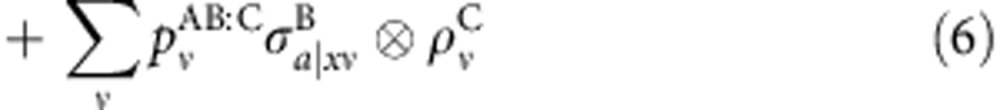


and


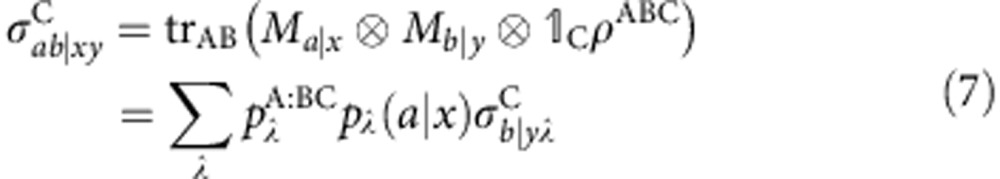



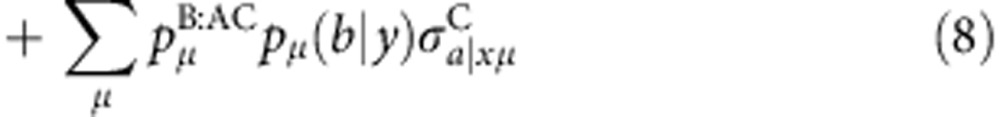



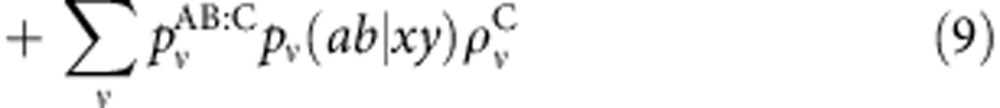


respectively.

Thus, the fact that the original state is biseparable imposes constraints on the observed assemblages. For instance, in equation (4), the dependence on the variables *a* and *x* is only through the distribution 
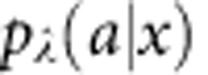
 and not through the quantum states. This is a typical instance of an unsteerable bipartite assemblage^10^. The assemblage in equation (5) satisfies two constraints: each conditional state is a separable state, and the dependence in *a* and *x* is due only to Charlie's system, and not Bob's. The assemblage in equation (6) is similar to the one in equation (5), only with Bob's and Charlie's roles exchanged. Thus, to test whether a given assemblage 
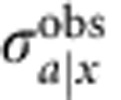
 has the form (4)–(6) consistent with having been produced by a biseparable state one could run the following program:

find assemblages


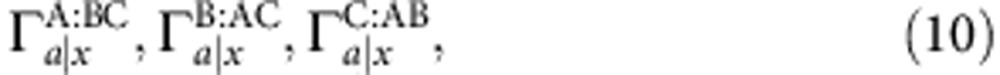


such that


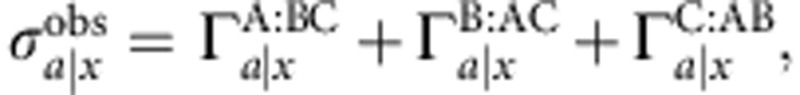



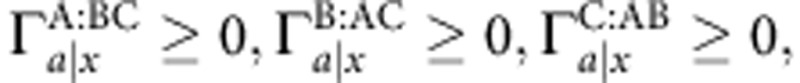



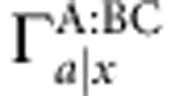
 is unsteerable,


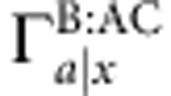
 is separable and unsteerable from A to B,


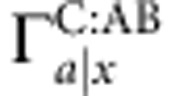
 is separable and unsteerable from A to C.

If no such triple of assemblages exists, then the underlying state was definitely not biseparable, and therefore genuine multipartite entangled. A problem with this method is that, apart from systems with dimension ≤6, testing separability is computationally demanding^34^. As we show in the Supplementary Notes 1 and 2, we can overcome this problem by considering approximations of the set of separable states, which relax the above program into a semidefinite program (SDP)^35^,^36^, for which efficient numerical methods exist.

A similar analysis can be made for the decomposition in equations (7)–(9), 17, 18 (see Supplementary Note 2) and other types of entanglement (see Supplementary Table 1). For instance, equation (7) refers to an assemblage that is unsteerable from A to C and equation (8) to one that is unsteerable from B to C. The assemblage (9) has two properties: it is unsteerable, and the probability distributions 
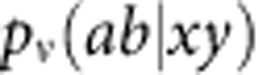
 must have quantum realizations, that is, must come from measurements on quantum states. Again, this last requirement is in general difficult to test. However, we can once again make use of relaxations of the set of quantum probability distributions^37^ to transform the program into an SDP.

All in all, for each semi-device-independent scenario the type of entanglement in the distributed state will impose constraints on the assemblages one could observe. These constraints allow the parties holding the trusted devices to determine whether this state must have had this type of entanglement (for example, if the observed data admit no decomposition of the form (4) or (7), then there exists no biseparable state that could explain it). Therefore, even not knowing the initial state or what type of measurements the untrusted parties performed, it is possible to discriminate the assemblages that were produced by states containing some type of entanglement.

Finally, in each case, the program can be seen as a membership test for the observed assemblage to be contained inside a convex set. It is always possible to certify that a point lies outside a convex set by finding a separating hyperplane between the set and the point. As we show in the Supplementary Note 2, in each case we can find the lagrange dual program to the set membership test, which always amounts to finding such a separating hyperplane. Such separating hyperplanes are precisely multipartite steering inequalities, which can alternatively be thought of as semi-device-independent entanglement witnesses. Thus, our method naturally generates steering inequalities, which can then be used as witnesses for multipartite entanglement.

### Practical considerations

Due to experimental errors and finite statistics, the experimentally observed data are not strictly compatible with any physical state and local measurements. In particular, all assemblages that exactly reproduce the experimental data in general do not satisfy the no-signalling constraint that 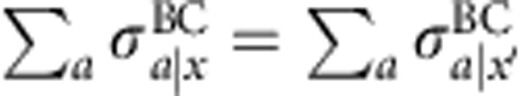 for *x*≠*x*′. Since the present methods are tailored to detect entanglement of physical states, we cannot use the observed data directly to test for the presence of entanglement.

We thus propose to proceed with the following steps: first, given the experimental data, generate a physical assemblage that best approximates it through, for instance, a maximum likelihood reconstruction method. Second, having obtained the best physical approximation to the actual data, use the SDP method discussed in the Supplementary Note 2 to check for any type of entanglement. This method also generates an inequality that is satisfied by all assemblages coming from states which do not have the type of entanglement tested for. Finally, check that the observed data violate this inequality.

### Example witnesses for GHZ and W states

As examples, we used our method to produce the following inequality that is satisfied by all assemblages of the form (4)–(6) (see also Supplementary 4):


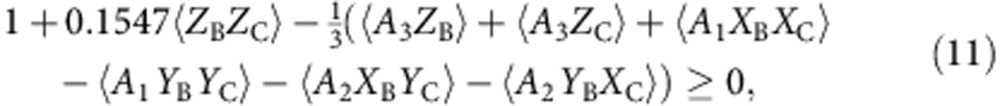


with *A*_*i*_ for *i*=1, 2, 3, being observables in Alice's system with outcomes labelled ±1 and *X*, *Y* and *Z* representing the Pauli operators. The Greenberger-Horne-Zeilinger (GHZ) state 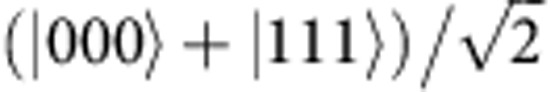 violates this inequality by 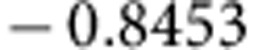 when Alice's measurements are also *X*, *Y* and *Z*, which numerical optimization suggests are the optimal choices for Alice.

In the case Alice and Bob perform untrusted measurements we have derived the following inequality, which is satisfied by assemblages of the form (7)–(10):


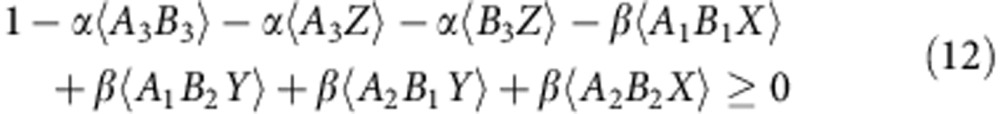


where *α*=0.1831 and *β*=0.2582, and similarly *B*_*i*_ for *i*=1, 2, 3 represent Bob's measurement, which we assume to have ±1 outcomes. The GHZ state achieves a violation 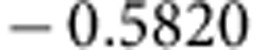 now when both Alice and Bob perform *X*, *Y* and *Z* measurements.

Similar inequalities for the W state, given by , with one or two untrusted parties are presented in the Supplementary Note 4.

We have also considered noisy versions of the GHZ and W states given by


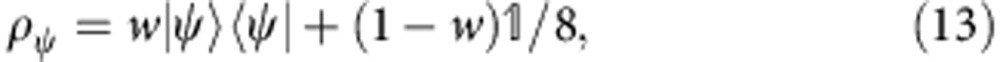


where 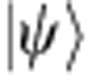 can be either the GHZ or the W state. We computed how much white noise can be added to these states until we are unable to detect genuine multipartite entanglement. Specifically, we quantify the minimum *w* for which our method guarantees that the states are genuinely multipartite entangled. The results are summarized in Table 1, together with the known bounds for standard entanglement tests^38^,^39^ and the device-independent case^23^. One can see that trusting some of the parties offers a significant advantage in terms of noise tolerance.

### Experimental violation of genuine tripartite steering witnesses

**Figure 2:**
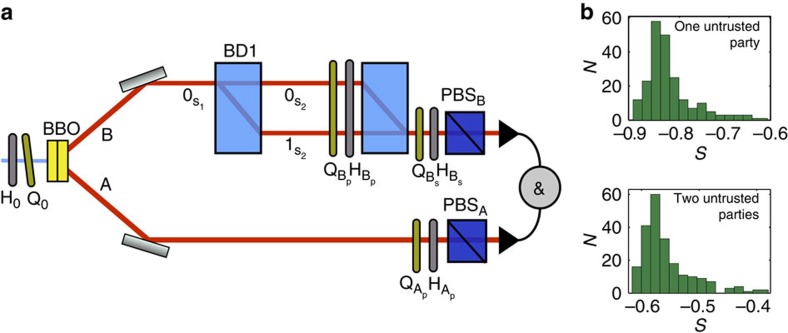
Experimental setup and results. (**a**) A 325 nm laser pumps two 1-mm long cross axis BBO crystals. Probabilistically, two photons are produced in the state (14) via parametric down conversion^42^. The polarization entangled state is a superposition of vertically polarized signal and idler beams produced in the first crystal and the horizontally polarized ones produced in the second crystal. Signal photons in B are sent to beam displacer BD1, which transmit vertical polarization and deviate horizontal polarization. This results in the production of a GHZ state after BD1, with two qubits encoded in the polarization of photons A and B, and one qubit encoded in the path of photon B. Photons in mode A are detected after polarization projection, which is done using the quarter-wave plate (QWP) Q_A_p__, half-wave plate (HWP) H_A_p__ and polarizing beam splitter PBSA. We perform a joint analysis of the polarization and path bases of photon B using the sequence of devices QWP Q_B_p__, HWP H_B_p__ beam displacer BD2, QWP Q_B_S__, HWP H_B_S__ and polarizing beam splitter PBS_B_. For given adjustments of the QWPs and HWPs, we perform one specific joint projection in the polarization and path basis. Since there is a coherent combination of spatial modes 0 and 1 in BD2, the measurement of the path of photon B is done by mapping the spatial qubit before BD2 into the polarization at the output of BD2. Even though the projection is made simultaneously for both qubits in this case, they are independent, or in other words, all combinations of projections are possible^40^,^41^. (**b**) Histograms obtained by computing the semi-device-independent entanglement witness from the experimental data (see main text and the Supplementary Note 5 for more details about the witness). We measured the value of each witness 215 independent times. The upper histogram is for the case of one untrusted party, resulting in the average value of −0.82 and s.d. of 0.05. The lower histogram is for the case of two untrusted parties, resulting in the average of −0.56 and s.d. of 0.04.

To illustrate the utility and efficiency of our approach, we use this technique to violate genuine multipartite steering witnesses in a real laboratory setting where one or two parties perform untrusted measurements. The experimental setup is shown in Fig. 2 and is set to produce a GHZ state encoded in the polarization and path degree of freedom of two photons^40^,^41^ with high fidelity. The experimental procedure starts by preparing photons in a state close to


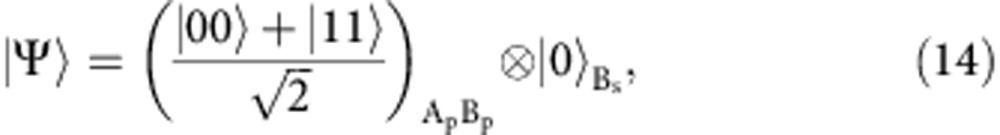


where A_p_ and B_p_ represent the polarization qubit of photons A and B, respectively, where 0 and 1 stand for horizontal and vertical polarization states, and B_s_ represents the spatial degree of freedom of photon B. To obtain a GHZ state, we couple the spatial degree of freedom with the polarization using beam displacer (BD1), which transforms 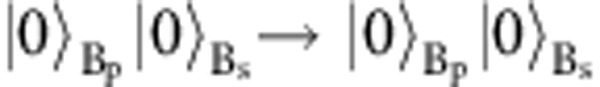 and 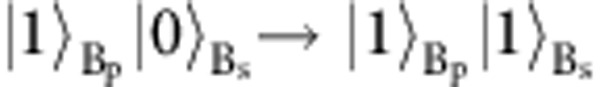. Once we obtain the desired state, every qubit is measured in the eigenstates of the three Pauli operators. For the polarization degrees of freedom, this is carried out using a quarter-wave plate (QWP), a half-wave plate (HWP) and a polarizing beam splitter or BD2, depending on the photon. For the spatial degrees of freedom, this is carried out using the interferometer described in Fig. 2 (refs 40, 41).

Although this experiment is tailored to produce a GHZ state and perform measurements corresponding to the Pauli operators, the analysis we perform on the experimental data makes no assumption about the state nor the untrusted measurements. We consider two cases, one where part A_p_ is untrusted and parts B_p_ and B_s_ hold the trusted devices, and another when parts B_p_ and B_s_ hold the untrusted devices and part A_p_ the trusted one. For the two cases, we follow the procedure described in section Practical considerations (using a least-squares optimization to provide physical assemblages), which provides inequalities of the form *S*≥0 (whose exact form can be found in the Supplementary Note 5) whose violation certify that the corresponding assemblages cannot be written in the biseparable form (4) or (7), respectively. We finally observe a violation of these inequalities by the experimental data (see Fig. 2b). We have performed each experiment (that is, measuring all correlators) 215 independent times, from which we calculate an average value of *S*=−0.82±0.05 for one untrusted party and *S*=−0.56±0.04 for two untrusted parties. This proves that there exists no biseparable tripartite state and measurements performed by the untrusted parties that could have generated the observed assemblages.

## Discussion

We have derived a method to detect multipartite entanglement when some of the apparatuses used in a quantum network are untrusted or uncharacterized. This method allows the detection of all kinds of entanglement in quantum networks where some of the observers use their measurement apparatuses simply as data lists. This scenario is experimentally less demanding than the nonlocality scenario, as it tolerates more noise, for instance. We have performed a proof-of-principle experiment demonstrating the existence of genuine tripartite entanglement, without any assumption on the source or the measurements being performed in some of the subsystems.

Our results provide a feasible test for multipartite entanglement in quantum networks and bridges the two well known cases of multipartite entanglement and multipartite Bell nonlocality. Moreover, the scenario considered is a natural generalization of bipartite quantum steering^10^ (see 28,30 for alternative definitions). Since steering has found applications in cryptographic protocols^11^,^12^, we believe that our results can be used as a starting point to define semi-device-independent cryptographic applications in future quantum networks.

## Additional information

**How to cite this article:** Cavalcanti, D. *et al*. Detection of entanglement in asymmetric quantum networks and multipartite quantum steering. *Nat. Commun*. 6:7941 doi: 10.1038/ncomms8941 (2015).

## Supplementary Material

Supplementary Information Supplementary Tables 1-2, Supplementary Notes 1-5

## Figures and Tables

**Table 1:** Critical robustness to white noise *w*.

**No. untr. meas.**	**0**	**1**	**2**	**3**
GHZ	3/7≈0.429	≈0.54	≈0.63	2/3≈0.67
W	≈0.479	≈0.57	≈0.67	≈0.72

We provide a comparison between the known bounds on critical robustness to white noise of the GHZ and W states above which genuine multipartite entanglement can be detected in four different scenarios: when no party is untrusted (that is, the standard entanglement scenario^38^,^39^), when 1 and 2 parties hold untrusted devices, for which we used the semi-device-independent method developed here, and when all devices are untrusted, that is, the device-independent case developed in ref. 23. In the Supplementary Table 2, we also display the bounds concerning the detection of (not necessarily genuine multipartite) entanglement in these states.
