# Supplementary material for: Detection of entanglement in asymmetric quantum networks and multipartite quantum steering
Source: Nat Commun. 2015 Aug 3;6:7941. doi: 10.1038/ncomms8941 (PMC4532875; doi:10.1038/ncomms8941)
Supplement: Supplementary Information — Supplementary Tables 1-2, Supplementary Notes 1-5 [file ncomms8941-s1.pdf]

Form of state	Unstrusted parties	Known objects	SDP
$\sum_\lambda p_\lambda \rho_\lambda^{\mathrm{A}} \otimes \rho_\lambda^{\mathrm{B}} \otimes \rho_\lambda^{\mathrm{C}}$	A	$\sigma_{a\|x}^{\mathrm{BC}}$	$\max \; p$ s.t $\sum_\mu D_\mu(a\|x)\sigma_\mu^{\mathrm{BC}} = \sigma_{a\|x}^{\mathrm{BC}} - p\,\mathrm{id}_{a\|x}^{\mathrm{BC}},$ $\left(\sigma_\mu^{\mathrm{BC}}\right)^{T_{\mathrm{B}}} \geq 0, \quad \sigma_\mu^{\mathrm{BC}} \geq 0.$ (1)
	A and B	$\sigma_{ab\|xy}^{\mathrm{C}}$	$\max \; p$ s.t. $\sum_{\mu,\lambda} D_\mu(a\|x) D_\lambda(b\|y)\sigma_{\mu\lambda}^{\mathrm{C}} = \sigma_{ab\|xy}^{\mathrm{C}} - p\,\mathrm{id}_{ab\|xy}^{\mathrm{C}},$ $\sigma_{\mu\lambda}^{\mathrm{C}} \geq 0.$ (2)
$\sum_\lambda p_\lambda \rho_\lambda^{\mathrm{A}} \otimes \rho_\lambda^{\mathrm{BC}}$	A	$\sigma_{a\|x}^{\mathrm{BC}}$	$\max \; p$ s.t $\sum_\mu D_\mu(a\|x)\sigma_\mu^{\mathrm{BC}} = \sigma_{a\|x}^{\mathrm{BC}} - p\,\mathrm{id}_{a\|x}^{\mathrm{BC}},$ $\sigma_\mu^{\mathrm{BC}} \geq 0.$ (3)
	B	$\sigma_{b\|y}^{\mathrm{AC}}$	$\max \; p$ s.t $\Gamma_{b\|y}^{\mathrm{AC}} = \sigma_{b\|y}^{\mathrm{AC}} - p\,\mathrm{id}_{b\|y}^{\mathrm{AC}},$ $\mathrm{tr}_{\mathrm{C}}\, \Gamma_{b\|y}^{\mathrm{AC}} = \sum_\mu D_\mu(b\|y)\sigma_\mu^{\mathrm{A}},$ $(\Gamma_{b\|y}^{\mathrm{AC}})^{T_{\mathrm{A}}} \geq 0, \quad \Gamma_{b\|y}^{\mathrm{AC}} \geq 0, \quad \sigma_\mu^{\mathrm{A}} \geq 0.$ (4)
	A and B	$\sigma_{ab\|xy}^{\mathrm{C}}$	$\max \; p$ s.t $\sum_\mu D_\mu(a\|x)\sigma_{b\|y,\mu}^{\mathrm{C}} = \sigma_{ab\|xy}^{\mathrm{C}} - p\,\mathrm{id}_{ab\|xy}^{\mathrm{C}},$ $\sigma_{b\|y,\mu}^{\mathrm{C}} \geq 0.$ (5)
	B and C	$\sigma_{bc\|yz}^{\mathrm{A}}$	$\max \; p$ s.t. $\sum_\mu D^{\mathrm{NS}}(bc\|yz,\mu)\sigma_\mu^{\mathrm{A}} = \sigma_{bc\|yz}^{\mathrm{A}} - p\,\mathrm{id}_{bc\|yz}^{\mathrm{A}}$ $\sum_\mu D^{\mathrm{NS}}(bc\|yz,\mu)\sigma_\mu^{\mathrm{A}} \in \mathcal{Q}_{\mathrm{A}}^{(k)}, \quad \sigma_\mu^{\mathrm{A}} \geq 0$ (6)
$\sum_\lambda p_\lambda^{\mathrm{A:BC}} \rho_\lambda^{\mathrm{A}} \otimes \rho_\lambda^{\mathrm{BC}}$ $+\sum_\lambda p_\lambda^{\mathrm{B:AC}} \rho_\lambda^{\mathrm{B}} \otimes \rho_\lambda^{\mathrm{AC}}$ $+\sum_\lambda p_\lambda^{\mathrm{AB:C}} \rho_\lambda^{\mathrm{AB}} \otimes \rho_\lambda^{C}$	A	$\sigma_{a\|x}^{\mathrm{BC}}$	$\max \; p$ s.t. $\Gamma_{a\|x}^{\mathrm{A:BC}} + \Gamma_{a\|x}^{\mathrm{B:AC}} + \Gamma_{a\|x}^{\mathrm{C:AB}} = \sigma_{a\|x}^{\mathrm{BC}} - p\,\mathrm{id}_{a\|x}^{\mathrm{BC}}$ $\Gamma_{a\|x}^{\mathrm{A:BC}} = \sum_\mu D_\mu(a\|x)\sigma_\mu^{\mathrm{BC}}, \quad \sigma_\mu^{\mathrm{BC}} \geq 0$ $\mathrm{tr}_{\mathrm{C}}\, \Gamma_{a\|x}^{\mathrm{B:AC}} = \sum_\mu D_\mu(a\|x)\sigma_\mu^{\mathrm{B}}, \quad \sigma_\mu^{\mathrm{B}} \geq 0,$ $\mathrm{tr}_{\mathrm{B}}\, \Gamma_{a\|x}^{\mathrm{C:AB}} = \sum_\mu D_\mu(a\|x)\sigma_\mu^{\mathrm{C}}, \quad \sigma_\mu^{\mathrm{C}} \geq 0,$ $\left(\Gamma_{a\|x}^{\mathrm{B:AC}}\right)^{T_{\mathrm{B}}} \geq 0, \quad \left(\Gamma_{a\|x}^{\mathrm{C:AB}}\right)^{T_{\mathrm{B}}} \geq 0.$ $\Gamma_{a\|x}^{\mathrm{B:AC}} \geq 0, \quad \Gamma_{a\|x}^{\mathrm{C:AB}} \geq 0, \quad \sum_a \Gamma_{a\|x}^{\mathrm{B:AC}} = \sum_a \Gamma_{a\|x'}^{\mathrm{B:AC}}.$ (7)
	A and B	$\sigma_{ab\|xy}^{\mathrm{C}}$	$\max \; p$ s.t. $\Pi_{ab\|xy}^{\mathrm{A:BC}} + \Pi_{ab\|xy}^{\mathrm{B:AC}} + \Pi_{ab\|xy}^{\mathrm{C:AB}} = \sigma_{ab\|xy}^{\mathrm{C}} - p\,\mathrm{id}_{ab\|xy}^{\mathrm{C}}$ $\Pi_{ab\|xy}^{\mathrm{A:BC}} = \sum_\mu D_\mu(a\|x)\sigma_{b\|y,\mu}^{\mathrm{C}}, \quad \sigma_{b\|y,\mu}^{\mathrm{C}} \geq 0$ $\Pi_{ab\|xy}^{\mathrm{B:AC}} = \sum_\mu D_\mu(b\|y)\sigma_{a\|x,\mu}^{\mathrm{C}}, \quad \sigma_{a\|x,\mu}^{\mathrm{C}} \geq 0$ $\Pi_{ab\|xy}^{\mathrm{C:AB}} = \sum_\mu D_\nu^{\mathrm{NS}}(ab\|xy)\sigma_\nu^{\mathrm{C}}, \quad \sigma_\nu^{\mathrm{C}} \geq 0$ $\Pi_{ab\|xy}^{\mathrm{C:AB}} \in \mathcal{Q}_{\mathrm{C}}^{(k)}, \quad \sum_b \sigma_{b\|y,\mu}^{\mathrm{C}} = \sum_b \sigma_{b\|y',\mu}^{\mathrm{C}}$ (8)

Supplementary Table I: **Collection of all SDP tests for the tripartite case.** All expressions with indices should be understood to hold for each value of the index. Various SDPs depend upon a parameter k, such that for larger values of k we obtain a better approximate characterisation of the set, and therefore a more stringent test. All programs are strictly feasible and are such that a negative optimal value $p^* < 0$ certifies that the assemblage has the corresponding type of entanglement. In this case the dual provides a semi-device-indepenent entanglement witness in the form of a steering inequality. An optimal solution $p^* \geq 0$ indicates that the assemblage is inside the corresponding set, i.e. that one cannot conclude that the state contains the desired type of entanglement.

		EW	SW		SW		DIEW	
no. untr.		0	1		2		3	
no. meas.		n/a	2	3	2	3	2	3
GHZ	ent.	$\frac{1}{5} = 0.2$ [1]	≈ 0.2613	≈ 0.2500	$\frac{1}{2} = 0.5$	$\frac{3}{7} \approx 0.4286$	$\frac{1}{2} = 0.5$ [5]	$\frac{1}{2} = 0.5$ [5]
	GME	$\frac{3}{7} \approx 0.4286$ [2]	≈ 0.6307	≈ 0.5420	$\frac{1}{\sqrt{2}} \approx 0.7071$	≈ 0.6322	$\frac{1}{\sqrt{2}} \approx 0.7071$ [6]	$\frac{2}{3} \approx 0.6667$ [6]
W	ent.	≈ 0.2096 [3]	$\frac{3}{11} \approx 0.2727$	≈ 0.2698	≈ 0.5765	≈ 0.4434	≈ 0.6442 [5]	≈ 0.6048 [5]
	GME	≈ 0.479 [4]	≈ 0.6440	≈ 0.5684	≈ 0.7218	≈ 0.6757	$3/4 = 0.75$ [6]	≈ 0.7158 [6]

Supplementary Table II: **Critical robustness to white noise w^* for different scenarios.** We provide a comparison between the critical robustness to white noise of the GHZ and W state, in the 4 different scenarios: All 3 parties with trusted devices, *i.e.* using an entanglement witness (EW), 2 or 1 parties with trusted devices, *i.e.* using a steering witness (SW) and no parties with trusted devices *i.e.* nonlocality (DIEW). We give critical values for both detecting entanglement and for detecting genuine multipartite entanglement ($\approx$ refers to numerical results).

Supplementary note 1

Characterising multipartite assemblages

Here we determine the constraints that different types of entanglement in the initial state ρ^{ABC} impose on the assemblages produced by untrusted measurements and define the corresponding sets that they characterise. We will first consider the question of whether or not there is any entanglement in the state, before moving on to entanglement in a given bipartition, for which there are a number of different cases, given the asymmetry of the scenario, and finish with the question of detecting genuine multipartite entanglement.

Case 1. Multipartite entanglement

Let us then start by considering a state $\rho^{\mathrm{A:B:C}}$ that is fully separable *i.e.*

$$\rho^{\mathrm{A:B:C}} = \sum_\lambda p_\lambda \rho_\lambda^{\mathrm{A}} \otimes \rho_\lambda^{\mathrm{B}} \otimes \rho_\lambda^{\mathrm{C}}, \tag{9}$$

where p_λ defines a probability distribution.

Case 1A. Multipartite entanglement with one untrusted party

We treat first the case where a single party, taken to be A, performs a set of untrusted measurements $\{M_{a|x}\}_{a,x}$ on her share of the state, providing the parties B and C with the assemblage

$$\begin{aligned}\sigma_{a|x}^{\mathrm{BC}} &= \mathrm{tr}_{\mathrm{A}}(M_{a|x} \otimes \mathbb{1}_{\mathrm{B}} \otimes \mathbb{1}_{\mathrm{C}} \rho^{\mathrm{A:B:C}}) \\ &= \sum_\lambda p(a|x,\lambda) \rho_\lambda^{\mathrm{B}} \otimes \rho_\lambda^{\mathrm{C}}.\end{aligned} \tag{10}$$

where $p(a|x,\lambda) = p_\lambda \, \mathrm{tr}(M_{a|x}\rho_\lambda^{\mathrm{A}})$. Notice first that the dependence of Bob and Charlie's assemblage on a and x comes only from the common pre-shared variable λ. This is a typical instance of an unsteerable assemblage, or, in other words, this is a local hidden state (LHS) model for the assemblage $\sigma_{a|x}^{\mathrm{BC}}$ [7]. Notice further that it is composed only by separable (unnormalised) states for B and C. Thus, in this case, testing for the presence of multipartite entanglement reduces to testing if the assemblage $\sigma_{a|x}^{\mathrm{BC}}$ is steerable and separable at the same time. This type of assemblages forms a set $\Sigma_{\mathrm{BC}}^{\mathrm{A:B:C}}$, given by

$$\Sigma_{\mathrm{BC}}^{\mathrm{A:B:C}} = \left\{ \sigma_{a|x}^{\mathrm{BC}} \middle| \sigma_{a|x}^{\mathrm{BC}} = \textstyle\sum_\mu D(a|x,\mu)\rho_\mu^{\mathrm{BC}}, \rho_\mu^{\mathrm{BC}} \in \mathrm{SEP} \right\} \tag{11}$$

where we have used the fact that any probability distribution $p(a|x,\lambda)$ can be written as a convex combination of deterministic ones $D(a|x,\mu)$, *i.e.* $p_\lambda(a|x) = \sum_\mu q(\mu|\lambda)D(a|x,\mu)$, and denote the set of (unnormalised) separable quantum states by SEP.

Case 1B. Multipartite entanglement with two untrusted parties

Now we consider that two of the parties, A and B, have untrusted measuring devices. In this case they prepare an assemblage for Charlie, which is given by

$$\begin{aligned}\sigma_{ab|xy}^{\mathrm{C}} &= \mathrm{tr}_{\mathrm{AB}}(M_{a|x} \otimes M_{b|y} \otimes \mathbb{1}_{\mathrm{C}} \rho^{\mathrm{A:B:C}}) \\ &= \sum_\lambda p_\lambda(ab|xy) \rho_\lambda^{\mathrm{C}}.\end{aligned} \tag{12}$$

Once again, since the only dependence of the assemblage on a, b, x and y is through λ this assemblage is also unsteerable. Moreover, because the set of probability distributions (also called a behaviour) $p_\lambda(ab|xy)$ arises from local measurements on a separable state it must be local, *i.e.* it can be written as $p_\lambda(ab|xy) = \sum_{\mu\nu} q(\mu\nu|\lambda)D(a|x,\mu)D(b|y,\nu)$ [8]. Therefore, the relevant set of assemblages $\Sigma_{\mathrm{C}}^{\mathrm{A:B:C}}$ is now given by

$$\Sigma_{\mathrm{C}}^{\mathrm{A:B:C}} = \left\{ \sigma_{ab|xy}^{\mathrm{C}} \middle| \sigma_{ab|xy}^{\mathrm{C}} = \textstyle\sum_{\mu\nu} D(a|x,\mu)D(b|y,\nu)\rho_{\mu\nu}^{\mathrm{C}}, \rho_{\mu\nu}^{\mathrm{C}} \geq 0 \right\}. \tag{13}$$

Case 2. Entanglement in a bipartition

Let us now consider the case where the state ρ^{ABC} is separable with respect to a single given bipartition. Choosing this partition to be A : BC we now consider states of the form

$$\rho^{\mathrm{A:BC}} = \sum_\lambda p_\lambda \rho_\lambda^{\mathrm{A}} \otimes \rho_\lambda^{\mathrm{BC}}. \tag{14}$$

Crucially, given the asymmetry of picking a bipartition as well as the asymmetry of picking the trusted party (or parties), we will see below that we have two inequivalent situations to consider, for both the cases of one or of two untrusted parties.

Case 2A. Entanglement in a bipartition with one untrusted party

When only one of the parties performs uncharacterised measurements the asymmetry of (14) leads to two different situations: (i) when the lone party A has the untrusted devices and (ii) when B (or equivalently C) does. In the first case Bob and Charlie's assemblage is given by

$$\begin{aligned}\sigma_{a|x}^{\mathrm{BC}} &= \mathrm{tr}_{\mathrm{A}}(M_{a|x} \otimes \mathbb{1}_{\mathrm{B}} \otimes \mathbb{1}_{\mathrm{C}} \rho^{\mathrm{A:BC}}) \\ &= \sum_\lambda p(a|x,\lambda) \rho_\lambda^{\mathrm{BC}},\end{aligned} \tag{15}$$

Once again the dependence of Bob and Charlie's assemblage on a and x comes only from the common variable

λ, and so this assemblage is unsteerable. In comparison to previously, the states distributed to Bob and Charlie are now arbitrary entangled states, and hence there is no additional structure that the decomposition imposes. The set $\Sigma_{\mathrm{BC}}^{\mathrm{A:BC}}$ defined by assemblages of the form (15) is therefore given by

$$\Sigma_{\mathrm{BC}}^{\mathrm{A:BC}} = \left\{\sigma_{a|x}^{\mathrm{BC}} \middle| \sigma_{a|x}^{\mathrm{BC}} = \textstyle\sum_\mu D(a|x,\mu)\rho_\mu^{\mathrm{BC}}, \rho_\mu^{\mathrm{BC}} \geq 0\right\}. \tag{16}$$

In the second case, where Bob is the one not trusting his measurements, Alice and Charlie are left with the following assemblage:

$$\begin{aligned}\sigma_{b|y}^{\mathrm{AC}} &= \mathrm{tr}_{\mathrm{B}}(\mathbb{1}_{\mathrm{A}} \otimes M_{b|y} \otimes \mathbb{1}_{\mathrm{C}}\rho^{\mathrm{A:BC}}) \\ &= \sum_\lambda p_\lambda \rho_\lambda^{\mathrm{A}} \otimes \sigma_{b|y,\lambda}^{\mathrm{C}},\end{aligned} \tag{17}$$

which is a fundamentally different situation. This assemblage now has two main features: (i) the only dependence on the variables b and y are due to Charlie's states. In other words, $\sigma_{b|y}^{\mathrm{AC}}$ is unsteerable from Bob to Alice but not necessarily from Bob to Charlie (note however that the no-signalling condition still holds from Bob to Charlie). This implies that if we trace out system C (or apply any quantum-to-classical map to it) the resulting assemblage for Alice alone will be unsteerable; (ii) it is composed by separable (unnormalised) states. The relevant set, $\Sigma_{\mathrm{AC}}^{\mathrm{A:BC}}$, is now given by

$$\begin{aligned}\Sigma_{\mathrm{AC}}^{\mathrm{A:BC}} = \Big\{\sigma_{b|y}^{\mathrm{AC}} \Big| &\,\mathrm{tr}_{\mathrm{C}}\,\sigma_{b|y}^{\mathrm{AC}} = \textstyle\sum_\mu D(b|y,\mu)\rho_\mu^{\mathrm{A}}, \\ &\rho_\mu^{\mathrm{A}} \geq 0, \sigma_{b|y}^{\mathrm{AC}} \in \mathrm{SEP}, \sum_b \sigma_{b|y}^{\mathrm{AC}} = \sum_b \sigma_{b|y'}^{\mathrm{AC}}\Big\}\end{aligned} \tag{18}$$

Case 2B. Entanglement in a bipartition with two untrusted parties

Again the asymmetry of the decomposition (14) leads to two different situations: In one the untrusted measurements are at A and B (or similarly A and C), whilst in the other they are at B and C. In the first case the assemblage obtained is given by

$$\begin{aligned}\sigma_{ab|xy}^{\mathrm{C}} &= \mathrm{tr}_{\mathrm{AB}}(M_{a|x} \otimes M_{b|y} \otimes \mathbb{1}_{\mathrm{C}}\rho^{\mathrm{A:BC}}) \\ &= \sum_\lambda p(a|x,\lambda)\sigma_{b|y,\lambda}^{\mathrm{C}}.\end{aligned} \tag{19}$$

This assemblage has only one main feature, that it may contain only steering from Bob to Charlie, and not from Alice to Charlie. It then defines the set $\Sigma_{\mathrm{C}}^{\mathrm{A:BC}}$ as

$$\begin{aligned}\Sigma_{\mathrm{C}}^{\mathrm{A:BC}} = \Big\{\sigma_{ab|xy}^{\mathrm{C}} \Big| &\,\sigma_{ab|xy}^{\mathrm{C}} = \textstyle\sum_\mu D(a|x,\mu)\sigma_{b|y,\mu}^{\mathrm{C}}, \\ &\sigma_{b|y,\mu}^{\mathrm{C}} \geq 0, \sum_b \sigma_{b|y,\mu}^{\mathrm{C}} = \sum_b \sigma_{b|y',\mu}^{\mathrm{C}}\Big\}\end{aligned} \tag{20}$$

In the second case the resulting assemblage is given by

$$\begin{aligned}\sigma_{bc|yz}^{\mathrm{A}} &= \mathrm{tr}_{\mathrm{BC}}(\mathbb{1}_{\mathrm{A}} \otimes M_{b|y} \otimes M_{c|z}\rho^{\mathrm{A:BC}}) \\ &= \sum_\lambda p(bc|yz,\lambda)\rho_\lambda^{\mathrm{A}}.\end{aligned} \tag{21}$$

Here, there are two main features: (i) this assemblage is unsteerable; (ii) The behaviour $p(bc|yz,\lambda)$ arises from local measurements on a possibly entangled state $\rho_\lambda^{\mathrm{BC}}$, it may contain nonlocal *quantum* correlations [8]. The final set we define is therefore $\Sigma_{\mathrm{A}}^{\mathrm{A:BC}}$, given by

$$\begin{aligned}\Sigma_{\mathrm{A}}^{\mathrm{A:BC}} = \Big\{\sigma_{bc|yz}^{\mathrm{A}} \Big| &\,\sigma_{bc|yz}^{\mathrm{A}} = \textstyle\sum_\lambda p(bc|yz,\lambda)\sigma_\lambda^{\mathrm{A}}, \\ &\sigma_\lambda^{\mathrm{A}} \geq 0, p(bc|yz,\lambda) \in \mathcal{Q}\Big\}\end{aligned} \tag{22}$$

where we have denoted by $\mathcal{Q}$ the set of probability distributions which can arise from local measurements on quantum states.

Case 3. Genuine multipartite entanglement

Let us now turn to the question of genuine multipartite entanglement (GME) detection. Genuine tripartite entangled states are the ones that can not be written as

$$\begin{aligned}\rho^{\mathrm{bisep}} = &\sum_\lambda p_\lambda^{\mathrm{A:BC}}\rho_\lambda^{\mathrm{A}} \otimes \rho_\lambda^{\mathrm{BC}} + \sum_\lambda p_\lambda^{\mathrm{B:AC}}\rho_\lambda^{\mathrm{B}} \otimes \rho_\lambda^{\mathrm{AC}} \\ &+ \sum_\lambda p_\lambda^{\mathrm{AB:C}}\rho_\lambda^{\mathrm{AB}} \otimes \rho_\lambda^{C},\end{aligned} \tag{23}$$

where $p_\lambda^{\mathrm{A:BC}}$, $p_\lambda^{\mathrm{B:AC}}$ and $p_\lambda^{\mathrm{AB:C}}$ are probability distributions. Our goal once again is to determine what constraints the form (23) imposes on the obtained assemblages, which will now follow straightforwardly given the analysis made before.

Case 3A. Genuine multipartite entanglement with one untrusted party

When Alice is the one holding the untrusted devices, Bob and Charlie's assemblage is given by

$$\sigma^{\mathrm{BC}}_{a|x} = \mathrm{tr}(M_{a|x} \otimes \mathbb{1}_{\mathrm{B}} \otimes \mathbb{1}_{\mathrm{C}} \rho^{\mathrm{bisep}}) = \underbrace{\sum_\lambda p^{\mathrm{A:BC}}_\lambda p(a|x,\lambda)\rho^{\mathrm{BC}}_\lambda}_{\Gamma^{\mathrm{A:BC}}_{a|x} \in \Sigma^{\mathrm{A:BC}}_{\mathrm{BC}}} + \underbrace{\sum_\lambda p^{\mathrm{B:AC}}_\lambda \rho^{\mathrm{B}}_\lambda \otimes \sigma^{\mathrm{C}}_{a|x,\lambda}}_{\Gamma^{\mathrm{B:AC}}_{a|x} \in \Sigma^{\mathrm{B:AC}}_{\mathrm{BC}}} + \underbrace{\sum_\lambda p^{\mathrm{AB:C}}_\lambda \sigma^{\mathrm{B}}_{a|x\lambda} \otimes \rho^{\mathrm{C}}_\lambda}_{\Gamma^{\mathrm{C:AB}}_{a|x} \in \Sigma^{\mathrm{C:AB}}_{\mathrm{BC}}} . \tag{24}$$

The terms $\Gamma^{\mathrm{A:BC}}_{a|x}$ and $\Gamma^{\mathrm{B:AC}}_{a|x}$ can be seen as assemblages having the same structure as the assemblages (15) and (17) respectively, while the assemblage $\Gamma^{\mathrm{C:AB}}_{a|x}$ is identical to $\Gamma^{\mathrm{B:AC}}_{a|x}$, except that the role of Bob and Charlie is interchanged.

Case 3B. Genuine multipartite entanglement with two untrusted parties

Consider now that Alice and Bob perform untrusted measurements, leading to:

$$\sigma^{\mathrm{C}}_{ab|xy} = \mathrm{tr}_{\mathrm{AB}}(M_{a|x} \otimes M_{b|y} \otimes \mathbb{1}_{\mathrm{C}} \rho^{\mathrm{bisep}}) = \underbrace{\sum_\lambda p^{\mathrm{A:BC}}_\lambda p(a|x,\lambda)\sigma^{\mathrm{C}}_{b|y,\lambda}}_{\Pi^{\mathrm{A:BC}}_{ab|xy} \in \Sigma^{\mathrm{A:BC}}_{\mathrm{C}}} + \underbrace{\sum_\lambda p^{\mathrm{B:AC}}_\lambda p(b|y,\lambda)\sigma^{\mathrm{C}}_{a|x,\lambda}}_{\Pi^{\mathrm{B:AC}}_{ab|xy} \in \Sigma^{\mathrm{B:AC}}_{\mathrm{C}}} + \underbrace{\sum_\lambda p^{\mathrm{AB:C}}_\lambda p(ab|xy,\lambda)\rho^{\mathrm{C}}_\lambda}_{\Pi^{\mathrm{C:AB}}_{ab|xy} \in \Sigma^{\mathrm{C:AB}}_{\mathrm{C}}} . \tag{25}$$

Again, the assemblages $\Pi^{\mathrm{A:BC}}_{ab|xy}$ and $\Pi^{\mathrm{B:AC}}_{ab|xy}$ are seen to have the same structure as (19), while the assemblage $\Pi^{\mathrm{C:AB}}_{ab|xy}$ has the same structure as (21), respectively.

Supplementary note 2

SDP tests and semi-device-independent entanglement witnesses

We have previously determined the constraints that each kind of entanglement imposes, and defined the corresponding sets of assemblages these constraints define. We now turn to the following practical question: given that we have observed a specific assemblage, can we test for a certain type of entanglement by checking whether or not the assemblage belongs to one of the previously defined sets?

Crucially, it turns out that all of the sets defined above are either specified solely in terms of positive semi-definite (PSD) constraints and linear matrix inequalities (LMIs), or can be approximated from the outside by a set with such a specification. Testing for membership inside such a set is an optimisation problem known as a semi-definite program (SDP), for which efficient numerical methods exist for the case of small systems, allowing for an answer to this question [9]. Moreover, due to the theory of duality, the dual SDP provides us with a semi-device-independent witness that allow us to certify the presence of the different types of entanglement solely from the knowledge of the assemblage. This is similar to the ideas of entanglement witnesses and Bell inequalities in the standard and fully device-independent scenarios

Deriving the SDP tests

In those cases where the the sets defined above are not specified solely in terms of PSD constraints and LMIs our strategy is to show that there exist suitable relaxations which are, that is to define bigger sets which are specified solely in terms of such constraints.

Working through in the order that they appeared, we shall consider each set in turn. The first set is $\Sigma^{\mathrm{A:B:C}}_{\mathrm{BC}}$, given in equation (11). It is the final constraint, $\rho^{\mathrm{BC}}_\mu \in \mathrm{SEP}$, that does not have the desired form, as the set of (unnormalised) separable states has in general a complicated structure. The one case where the set in fact has a simple characterisation is if the dimensions satisfy $d_{\mathrm{B}} d_{\mathrm{C}} \leq 6$, in which case the set of separable states is exactly the set of states positive under partial transposition (PPT) [10]. In this simple case we can rewrite $\rho^{\mathrm{BC}}_\mu \in \mathrm{SEP}$ as $\left(\rho^{\mathrm{BC}}_\mu\right)^{\mathrm{T_B}} \geq 0$, where $\mathrm{T_B}$ denotes the partial transposition with respect to system B. Since this operation is a linear map (on the state), this is now a PSD constraint, and the set is in fact in the desired form.

In all other dimensions, we can use the relaxation of the separable states to those that have a k-symmetric

PPT extension [11]. That is, we define the set $\mathrm{SYM}_{\mathrm{BC}}^{(k)}$

$$\mathrm{SYM}_{\mathrm{BC}}^{(k)} = \Big\{\rho^{\mathrm{BC}}\Big|\rho^{\mathrm{BC}} = \mathrm{tr}_{\mathrm{B}_2\cdots\mathrm{B}_k}\,\rho^{\mathrm{B}_1\cdots\mathrm{B}_k\mathrm{C}}, \\ S_{ij}\rho^{\mathrm{B}_1\cdots\mathrm{B}_k\mathrm{C}}S_{ij}^{\dagger} = \rho^{\mathrm{B}_1\cdots\mathrm{B}_k\mathrm{C}} \quad \forall i\neq j, \\ \left(\rho^{\mathrm{B}_1\cdots\mathrm{B}_k\mathrm{C}}\right)^{T_{\mathrm{C}}} \geq 0, \rho^{\mathrm{B}_1\cdots\mathrm{B}_k\mathrm{C}} \geq 0\Big\} \quad (26)$$

where S_{ij} is the swap operator between B_i and B_j. This demands that ρ^{BC} can be extended to a state with k Bobs, which is symmetric under interchange and PPT, such that the reduced state of a single Bob and Charlie is the original state. Such sets are all specified in terms of PSD constraints and LMIs, and converge to the set of separable states as $k\to\infty$ [11, 12]. For the case $k=1$ it also reduces to the set of PPT state. We thus define the sequence of relaxations

$$\Sigma_{\mathrm{BC}}^{\mathrm{A:B:C}(k)} = \Big\{\sigma_{a|x}^{\mathrm{BC}}\Big|\sigma_{a|x}^{\mathrm{BC}} = \textstyle\sum_{\mu}D(a|x,\mu)\rho_{\mu}^{\mathrm{BC}}, \\ \rho_{\mu}^{\mathrm{BC}}\in\mathrm{SYM}_{\mathrm{BC}}^{(k)}\Big\}, \quad (27)$$

which now have the desired structure for each k.

Moving on, the set $\Sigma_{\mathrm{C}}^{\mathrm{A:B:C}}$ given in (13) already has the desired structure. This is also true for the set $\Sigma_{\mathrm{BC}}^{\mathrm{A:BC}}$ defined in (16). The set $\Sigma_{\mathrm{AC}}^{\mathrm{A:BC}}$ (18) contains the requirement that $\sigma_{b|y}^{\mathrm{AC}}\in\mathrm{SEP}$, which is dealt with in exactly the same way as above. Thus we define the relaxed set

$$\Sigma_{\mathrm{AC}}^{\mathrm{A:BC}(k)} = \Big\{\sigma_{b|y}^{\mathrm{AC}}\Big|\,\mathrm{tr}_{\mathrm{C}}\,\sigma_{b|y}^{\mathrm{AC}} = \textstyle\sum_{\mu}D(b|y,\mu)\rho_{\mu}^{\mathrm{A}}, \\ \rho_{\mu}^{\mathrm{A}}\geq 0, \sigma_{b|y}^{\mathrm{AC}}\in\mathrm{SYM}_{\mathrm{AC}}^{(k)}, \sum_b \sigma_{b|y}^{\mathrm{AC}} = \sum_b \sigma_{b|y'}^{\mathrm{AC}}\Big\} \quad (28)$$

The set $\Sigma_{\mathrm{C}}^{\mathrm{A:BC}}$ given in (20) has the desired structure.

Finally, the set $\Sigma_{\mathrm{C}}^{\mathrm{C:AB}}$ (which is has the same structure as (22)) is less straightforward because of the constraint $p(ab|xy,\lambda)\in\mathcal{Q}$, *i.e.* that the behaviours $p(ab|xy,\lambda)$ should have a quantum realisation. As in the nonlocality scenario of deciding if a behaviour has a quantum realisation, the exact answer to this problem is in general intractable. However, we can use the idea introduced in [13] (see also [14]) to obtain a semi-definite relaxation. The basic idea is to apply the method of the NPA hierarchy [15] only to the untrusted devices, whilst leaving Charlie quantum, using also the fact that the state is separable on the AB : C partition. We thus relax to $\Pi_{ab|xy}^{\mathrm{C:AB}}\in\mathcal{Q}_{\mathrm{C}}^{(k)}$, where $\mathcal{Q}_{\mathrm{C}}^{(k)}$ is defined by

$$\mathcal{Q}_{\mathrm{C}}^{(k)} = \Big\{\sigma_{ab|xy}^{\mathrm{C}}\Big|\Gamma_{\mathrm{C}}^{(k)}\geq 0, \left(\Gamma_{\mathrm{C}}^{(k)}\right)^{\mathrm{T_C}}\geq 0 \\ \mathrm{tr}\left(G_j\Gamma_{\mathrm{C}}^{(k)}\right) = \mathrm{tr}\left(h_j\sigma_{ab|xy}^{\mathrm{C}}\right)\forall j\Big\} \quad (29)$$

for some sets of operators $\{G_j^{(k)}\}$ and $\{h_j^{(k)}\}$, which encode the constraints that arise in the original NPA hierarchy [15], coming from (i) orthogonality of measurement outcomes, and (ii) commutivity of Alices and Bob measurements. Also, we can apply the idea from [13] and impose that the matrices $\Gamma_{\mathrm{C}}^{(k)}$ are positive under partial transposition of Charlie (the trusted party), as a relaxation of the separability criteria across the bipartition. The main difference with the NPA approach is that whereas previously the elements of the matrix $\Gamma^{(k)}$ were complex numbers with certain ones equal to the nonlocal behaviour, now one should think of the elements as matrices (of the dimension of Charlie), with certain ones equal to members of the assemblage.

Last, in order to be able to impose semidefinite constraints to the set $\Sigma_{\mathrm{C}}^{\mathrm{C:AB}}$ we need to constrain the number of terms in the summation in λ. We do this by noticing that any quantum behaviour can be written as a convex combination of extremal non-signalling behaviours $D^{\mathrm{NS}}(bc|yz,\nu)$ [8].

Given the above, set $\Sigma_{\mathrm{A}}^{\mathrm{C:AB}}$ is relaxed to

$$\Sigma_{\mathrm{C}}^{\mathrm{C:AB}(k)} = \Big\{\sigma_{ab|xy}^{\mathrm{C}}\Big|\sigma_{ab|xy}^{\mathrm{C}} = \textstyle\sum_{\nu}D^{\mathrm{NS}}(ab|xy,\nu)\sigma_{\nu}^{\mathrm{C}}, \\ \sigma_{ab|xy}^{\mathrm{C}}\in\mathcal{Q}_{\mathrm{C}}^{(k)}\Big\}. \quad (30)$$

Having found appropriate relaxations of all of the sets which we wish to consider, we can now straightforwardly write down an approximate optimisation problem in the form of an SDP that needs to be solved to check for the desired type of entanglement in each given scenario. Let us describe explicitly the approximate test that checks for the existence of a decomposition of the form (25), *i.e.* that checks for genuine multipartite entanglement with two untrusted parties. We provide all the other SDP tests for the other decompositions described above in Supplementary Table I.

In (25) we see that we have to find 3 assemblages, each contained in a different set, with each set either in a form directly usable, or for which we just gave an outer approximation above. Thus, by introducing the *maximally mixed assemblage* $\mathrm{id}_{ab|xy}^{\mathrm{C}} = \frac{1}{m_{\mathrm{A}}m_{\mathrm{B}}}\mathbb{1}_{\mathrm{C}}/d_{\mathrm{C}}$ we arrive at the following SDP test for genuine multipartite entanglement with 2 untrusted parties

$$\begin{aligned} \max\quad & p \\ \text{s.t.}\quad & \Pi_{ab|xy}^{\mathrm{A:BC}} + \Pi_{ab|xy}^{\mathrm{B:AC}} + \Pi_{ab|xy}^{\mathrm{C:AB}} = \sigma_{ab|xy}^{\mathrm{obs}} - p\,\mathrm{id}_{ab|xy}^{\mathrm{C}} \\ & \Pi_{ab|xy}^{\mathrm{A:BC}}\in\Sigma_{\mathrm{C}}^{\mathrm{A:BC}}, \Pi_{ab|xy}^{\mathrm{B:AC}}\in\Sigma_{\mathrm{C}}^{\mathrm{B:AC}}, \\ & \Pi_{ab|xy}^{\mathrm{C:AB}}\in\Sigma_{\mathrm{C}}^{\mathrm{C:AB}(k)} \end{aligned} \quad (31)$$

where $\sigma_{ab|xy}^{\mathrm{obs}}$ is the observed assemblage of Charlie. Since $\mathrm{id}_{ab|xy}^{\mathrm{C}}$ is clearly contained in all 3 sets, being producible from the maximally mixed state, a sufficiently large negative p will always be a solution, hence the SDP is strictly feasible. A strictly negative optimal solution $p^*<0$ certifies that $\sigma_{ab|xy}^{\mathrm{obs}}$ being measured does not have the desired decomposition, i.e. that the state is genuinely multipartite entangled. On the other hand an optimal

value $p^* = 0$ indicates that a decomposition can be found. Note however that in this case, given the relaxation of the problem, one is not able to conclude anything regarding the separability of the state. One can take the parameter k larger to obtain a better approximation to the original problem.

Semi device-independent entanglement witnesses

The dual of the SDP (31) is also readily written down [9], and is given by

$$\begin{aligned}\min \quad & \mathrm{tr}\sum_{abxy} F_{ab|xy}\sigma^{\mathrm{obs}}_{ab|xy} \\ \text{s.t.} \quad & \mathrm{tr}\sum_{abxy} F_{ab|xy}\sigma^{\mathrm{C}}_{ab|xy} \geq 0 \\ & \qquad \forall\ \sigma^{\mathrm{C}}_{ab|xy} \in \Sigma^{\mathrm{A:BC}}_{\mathrm{C}} \cup \Sigma^{\mathrm{B:AC}}_{\mathrm{C}} \cup \Sigma^{\mathrm{C:AB}(k)}_{\mathrm{C}} \\ & \mathrm{tr}\sum_{abxy} F_{ab|xy}\mathrm{id}^{\mathrm{C}}_{ab|xy} = 1\end{aligned} \tag{32}$$

which is seen to constitute a witness for genuine multipartite entanglement. That is, the dual provides a set of operators $\{F_{ab|xy}\}_{abxy}$ such that the linear functional $\beta = \sum_{abxy} F_{ab|xy}\sigma_{ab|xy}$ is greater than zero for all assemblages which arise from measurements on a bi-separable state. An observed value $\beta^{\mathrm{obs}} < 0$ thus provides a witness which certifies the genuine multipartite entanglement of the state in a semi-device-independent manner. The final condition, $\mathrm{tr}\sum_{abxy} F_{ab|xy}\mathrm{id}^{\mathrm{C}}_{ab|xy} = 1$ is a convention, which simply defines an overall scale for the witness.

More generally, the dual of each SDP in Supplementary Table I provides witness operators $\{F_{a|x}\}_{ax}$, for the case of Alice untrusted, or $\{F_{ab|xy}\}_{abxy}$, for the case of Alice and Bob untrusted (or a permutation of the parties) which constitute a semi-device-independent entanglement witnesses of the form

$$\begin{aligned}&\mathrm{tr}\sum_{ax} F_{a|x}\sigma_{a|x} \geq 0 \quad \forall \sigma_{a|x} \in \Sigma \\ &\mathrm{tr}\sum_{abxy} F_{ab|xy}\sigma_{ab|xy} \geq 0 \quad \forall \sigma_{ab|xy} \in \Sigma'\end{aligned} \tag{33}$$

with corresponding violations $\beta^{\mathrm{obs}} = \mathrm{tr}\sum_{ax} F_{a|x}\sigma^{\mathrm{obs}}_{a|x} < 0$ or $\beta^{\mathrm{obs}} = \mathrm{tr}\sum_{ax} F_{ab|xy}\sigma^{\mathrm{obs}}_{ab|xy} < 0$ respectively, where Σ and Σ' are sets, or union of sets (depending upon the type of entanglement one is checking for), as defined above.

Finally, we note that it is possible to put these witnesses into two more friendly forms in the case of binary measurement outcomes, so-called *observable* and *coefficient* forms. Starting with the observable form, we use the definition of the observed assemblage, and introduce the observables $A_x = M_{0|x} - M_{1|x}$ to arrive at

$$\begin{aligned}\mathrm{tr}\sum_{ax} F_{a|x}\sigma^{\mathrm{obs}}_{a|x} &= \mathrm{tr}\sum_{ax} M_{a|x}\otimes F_{a|x}\rho \\ &= \tfrac{1}{2}\,\mathrm{tr}\sum_{ax}(\mathbb{1}_{\mathrm{A}} + (-1)^a A_x)\otimes F_{a|x}\rho \\ &= \mathrm{tr}\left(\mathbb{1}_{\mathrm{A}}\otimes \tfrac{1}{2}\sum_{ax} F_{a|x} + \sum_x A_x \otimes \tfrac{1}{2}\sum_a (-1)^a F_{a|x}\right)\rho \\ &= \mathrm{tr}\left(\mathbb{1}_{\mathrm{A}}\otimes J_{\emptyset} + \sum_x A_x\otimes J_x\right)\rho\end{aligned} \tag{34}$$

for the case of one untrusted party, where we have defined the observables $J_{\emptyset} = \frac{1}{2}\sum_{ax} F_{a|x}$ and $J_x = \frac{1}{2}\sum_a (-1)^a F_{ab|xy}$ for Bob and Charlie. For the coefficient form we further expand these matrices in the (complete) basis of Pauli operators, namely,

$$J_{\emptyset} = \sum_{yz} f_{0yz}\sigma^y\otimes\sigma^z, \qquad J_x = \sum_{yz} f_{xyz}\sigma^y\otimes\sigma^z \tag{35}$$

where $\sigma^0 = \mathbb{1}$, $\sigma^1 = X$, $\sigma^2 = Y$ and $\sigma^3 = Z$ and denoting $A_0 = \mathbb{1}$ then we arrive at the compact form

$$\mathrm{tr}\sum_{ax} F_{a|x}\sigma^{\mathrm{obs}}_{a|x} = \mathrm{tr}\left(\sum_{xyz}(f_{xyz}A_x\otimes\sigma^y\otimes\sigma^z)\rho\right) \tag{36}$$

For the case of two untrusted parties, an analogous but longer calculation gives for the observable form

$$\begin{aligned}&\mathrm{tr}\sum_{abxy} F_{ab|xy}\sigma^{\mathrm{obs}}_{ab|xy} \\ &\quad = \mathrm{tr}\Big(\mathbb{1}_{\mathrm{A}}\otimes\mathbb{1}_{\mathrm{B}}\otimes K_{\emptyset} + \sum_x A_x\otimes\mathbb{1}_{\mathrm{B}}\otimes K_x \\ &\qquad + \sum_y \mathbb{1}_{\mathrm{A}}\otimes B_y\otimes K'_y + \sum_{xy} A_x\otimes B_y\otimes K_{xy}\Big)\rho\end{aligned} \tag{37}$$

where we have introduced the observables B_y for Bob, as well as the observables $K_{\emptyset} = \frac{1}{4}\sum_{abxy} F_{ab|xy}$, $K_x = \frac{1}{4}\sum_{aby}(-1)^a F_{ab|xy}$, $K'_y = \frac{1}{4}\sum_{abx}(-1)^b F_{ab|xy}$ and $K_{xy} = \frac{1}{4}\sum_{ab}(-1)^{a+b}F_{ab|xy}$ for the trusted party Charlie, that need to be measured in the corresponding configurations, given above. For the coefficient form, again by expanding these matrices in the complete basis of Pauli matrices,

$$\begin{aligned}K_{\emptyset} &= \sum_z f_{00z}\sigma^z, & K_x &= \sum_z f_{x0z}\sigma^z \\ K'_y &= \sum_z f_{0yz}\sigma^z, & K_{xy} &= \sum_z f_{xyz}\sigma^z\end{aligned} \tag{38}$$

and by denoting $B_0 = \mathbb{1}$, we obtain the analogous compact form as previously,

$$\mathrm{tr}\sum_{abxy} F_{ab|xy}\sigma^{\mathrm{obs}}_{ab|xy} = \mathrm{tr}\left(\sum_{xyz}(f_{xyz}A_x\otimes B_y\otimes\sigma^z)\rho\right) \tag{39}$$

Supplementary note 3

Generalisation to more parties

We have presented our main results in the tripartite case. Notice however that the same procedure can readily be followed to derive SDPs to test the presence of different kinds of entanglement for general N-partite systems.

First of all one specifies the scenarios by fixing (i) a particular type of entanglement and (ii) the pattern of trusted and untrusted parties. The entanglement can be chosen arbitrarily, for example one may ask that the state is not fully separable, be separable across a given number of fixed bipartitions, or be a convex combination of states separable over a given number of partitions (but not necessarily fixed). The patten may also be chosen arbitrarily, ranging from all but one party trusted, to all but one untrusted.

Given the specification, one then enumerates the list of properties which the corresponding assemblages have. These properties will fall into two classes - those which impose constraints which are directly applicable, *i.e.* are in the form of PSD constraints and LMIs, and those which are not. As in the tripartite case, the objective is then to relax the non-directly applicable constraints to find an approximate SDP test.

The main difficultly in our approach is that as the number of parties increases, and the local dimension of the Hilbert space, we expect that the difficulty of the problem will grow to the point where current numerical techniques are unable to solve efficiently the tests. For example, one class of constraints that will arise is that multipartite assemblages will need to have quantum realisations. In principle such a constraint can still be imposed by applying the NPA hierarchy [15] to the untrusted devices, however in the multipartite setting this soon becomes intractable. Alternatively, one may have constraints that a multipartite quantum state is separable. One can again relax this using the generalisation of the k-shareability condition [12].

In summary, the approach presented here is most suitable to scenarios involving relatively small numbers of parties, where it provides powerful tests for multipartite entanglement (and explicitly provides witnesses in each case). This is however expected as this is also the case in standard entanglement detection techniques [16] (due to the increase of the Hilbert space dimension) and in the fully device-independent approach [8] (due to the number of the space of local probability distributions).

Supplementary note 4

Examples: GHZ and W states

In order to demonstrate the usefulness of our previous characterisation we apply the above SDP to two exemplary genuine multipartite states, namely the GHZ and W states. More specifically we are interested in how much white noise can be added to these states until our method fails to detect either entanglement or GME, *i.e.* we want find the minimum w, denoted by w^*, allowing us to detect either entanglement or GME in the states

$$\begin{aligned}\rho_{\text{GHZ}} &= w|\text{GHZ}\rangle\langle\text{GHZ}| + (1-w)\mathbb{1}/8;\\ \rho_W &= w|W\rangle\langle W| + (1-w)\mathbb{1}/8, \end{aligned}\tag{40}$$

where $|\text{GHZ}\rangle = (|000\rangle + |111\rangle)/\sqrt{2}$ and $|W\rangle = (|001\rangle + |010\rangle + |100\rangle)/\sqrt{3}$. Supplementary Table II gives a summary of the results, in terms of the numbers provided by our methods and a comparison to what was known regarding entanglement witnesses and Bell inequalities. All results were obtained using CVX [9] for MATLAB to solve the SDP, and the optimisation toolbox to numerically search for the best choices of measurements for Alice (and Bob). Since such a search over measurements choices provides no guarantee that the global optimum is obtained, all results constitute upper bounds. However, all of our numerical evidence suggests that the values obtained cannot be improved.

As we can see the values of w^* lies in between the bound for entanglement, where the largest number of assumptions are made, and the bound from nonlocality, where no assumptions are made. Furthermore, as one would expect, stronger bounds are possible with 2 parties trust their devices compared to the case of only 1.

We end by presenting the steering witnesses we obtain in the above for the GHZ and W states that certify genuine tripartite entanglement in a semi-device-independent fashion.

Starting with the GHZ state and the case of two untrusted parties (and three measurements), the optimal witness is

$$\begin{aligned}1 - \alpha\langle A_2B_2\rangle - \alpha\langle A_2Z\rangle - \alpha\langle B_2Z\rangle - \beta\langle A_0B_0X\rangle \\ + \beta\langle A_0B_1Y\rangle + \beta\langle A_1B_0Y\rangle + \beta\langle A_1B_1X\rangle \geq 0\end{aligned}\tag{41}$$

where $\alpha = 0.1831$ and $\beta = 0.2582$, and the pure GHZ state achieves a violation $-0.5821 \ngeq 0$. For the case of the GHZ state and only a single untrusted party, the witness is

$$\begin{aligned}1 + 0.1547\langle Z_\text{B}Z_\text{C}\rangle - \tfrac{1}{3}\big(\langle A_2Z_\text{B}\rangle + \langle A_2Z_\text{C}\rangle + \langle A_0X_\text{B}X_\text{C}\rangle \\ - \langle A_0Y_\text{B}Y_\text{C}\rangle - \langle A_1X_\text{B}Y_\text{C}\rangle - \langle A_1Y_\text{B}X_\text{C}\rangle\big) \geq 0\end{aligned}\tag{42}$$

with the pure GHZ state now achieving a violation of $-0.8453 \ngeq 0$. Interestingly, we note first that the structure of both witnesses is the same, the only difference being in the coefficients. Furthermore the only terms which appear are those which arise from the stabiliser relations of the GHZ state.

Moving on to the W state, for two untrusted parties the optimal witness is

$$\begin{aligned} &1 + 0.2517\big(\langle A_2\rangle + \langle B_2\rangle\big) + 0.3520\langle Z\rangle - 0.1112\big(\langle A_0 X\rangle \\ &+ \langle A_1 Y\rangle + \langle B_0 X\rangle + \langle B_1 Y\rangle\big) + 0.1296\big(\langle A_2 Z\rangle + \langle B_2 Z\rangle\big) \\ &- 0.1943\big(\langle A_0 B_0\rangle + \langle A_1 B_1\rangle\big) + 0.2277\langle A_2 B_2\rangle \\ &- 0.1590\big(\langle A_0 B_0 Z\rangle + \langle A_1 B_1 Z\rangle\big) + 0.2228\langle A_2 B_2 Z\rangle \\ &-0.2298\big(\langle A_0 B_2 X\rangle + \langle A_1 B_2 Y\rangle + \langle A_2 B_0 X\rangle + \langle A_2 B_1 Y\rangle\big) \geq 0 \end{aligned} \quad (43)$$

and the pure W state obtains the violation $-0.4803 \ngeq 0$. For one untrusted party the witness is

$$\begin{aligned} &1 + 0.4405\big(\langle Z_B\rangle + \langle Z_C\rangle\big) - 0.0037\langle Z_B Z_C\rangle - 0.1570\big(\langle X_B X_C\rangle \\ &+ \langle Y_B Y_C\rangle + \langle A_2 X_B X_C\rangle + \langle A_2 Y_B Y_C\rangle\big) + 0.2424\big(\langle A_2\rangle \\ &+ \langle A_2 Z_B Z_C\rangle\big) + 0.1848\big(\langle A_2 Z_B\rangle + \langle A_2 Z_C\rangle\big) - 0.2533\big(\langle A_0 X_B\rangle \\ &+ \langle A_0 X_C\rangle + \langle A_1 Y_B\rangle + \langle A_1 Y_C\rangle + \langle A_0 X_B Z_C\rangle + \langle A_0 Z_B X_C\rangle \\ &+ \langle A_1 Y_B Z_C\rangle + \langle A_1 Z_B Y_C\rangle\big) \geq 0 \end{aligned} \quad (44)$$

with he pure W state achieving the violation $-0.7594 \ngeq 0$. Again, we note that structurally the witnesses are the same in the case of one and two untrusted parties.

Supplementary note 5

Experimental inequalities

In this section we give the semi-device independent entanglement witnesses that were used to optimally certify the presence of genuine multipartite entanglement of the GHZ state. Starting with the case of one untrusted party, in coefficient form the inequality is given by

$$f_{xy0} = \begin{pmatrix} 1.0000 & -0.0183 & 0.1079 & 0.0130 \\ 0.1522 & 0.1518 & -0.0734 & 0.0251 \\ -0.2870 & -0.1125 & -0.1229 & 0.0189 \\ -0.0658 & 0.0095 & -0.0151 & -0.2142 \end{pmatrix},$$

$$f_{xy1} = \begin{pmatrix} 0.2096 & 0.0941 & 0.1565 & 0.0077 \\ 0.0633 & 0.3006 & -0.0195 & 0.0132 \\ -0.0388 & -0.0133 & -0.3040 & -0.0033 \\ 0.0614 & 0.0040 & -0.0389 & -0.0387 \end{pmatrix},$$

$$f_{xy2} = \begin{pmatrix} -0.1487 & -0.1841 & 0.0862 & -0.0061 \\ -0.1839 & 0.0323 & 0.2801 & 0.0028 \\ 0.1036 & 0.2920 & -0.0423 & 0.0071 \\ 0.0066 & 0.0683 & 0.0277 & 0.0242 \end{pmatrix}, \quad (45)$$

$$f_{xy3} = \begin{pmatrix} 0.0189 & 0.0114 & 0.0363 & -0.2187 \\ -0.0259 & -0.0144 & 0.0255 & -0.0204 \\ -0.0714 & -0.0027 & 0.0099 & 0.0505 \\ -0.0189 & 0.1263 & -0.2037 & 0.0145 \end{pmatrix},$$

where x labels the rows and y the columns. For two untrusted parties the inequality is given by

$$f_{xy0} = \begin{pmatrix} 1.0000 & 0.2375 & -0.2613 & 0.0023 \\ 0.0026 & -0.0041 & -0.0055 & 0.0241 \\ -0.0039 & -0.0055 & -0.0114 & 0.0291 \\ -0.0012 & 0.0014 & 0.0032 & -0.1515 \end{pmatrix},$$

$$f_{xy1} = \begin{pmatrix} 0.0041 & -0.0033 & -0.0022 & 0.0275 \\ 0.1673 & 0.2676 & -0.0317 & 0.0044 \\ 0.1710 & -0.0015 & -0.2737 & -0.0072 \\ 0.0009 & 0.0059 & 0.0004 & 0.0001 \end{pmatrix},$$

$$f_{xy2} = \begin{pmatrix} -0.0063 & -0.0091 & 0.0053 & 0.0106 \\ -0.1669 & -0.0004 & 0.2651 & 0.0073 \\ 0.1675 & 0.2569 & -0.0307 & 0.0036 \\ 0.0019 & 0.0161 & 0.0127 & 0.0004 \end{pmatrix}, \quad (46)$$

$$f_{xy3} = \begin{pmatrix} -0.0013 & 0.0054 & 0.0011 & -0.1523 \\ 0.0024 & 0.0089 & 0.0053 & 0.0002 \\ 0.0023 & 0.0053 & 0.0047 & 0.0001 \\ -0.1527 & -0.0050 & -0.0085 & -0.0014 \end{pmatrix}.$$

Supplementary references

[1] W. Dür and J. Cirac, Phys. Rev. A **61**, 042314 (2000).
[2] O. Gühne and M. Seevinck, New J. Phys. **12**, 053002 (2010).
[3] S. Szalay, Phys. Rev. A **83**, 062337 (2011).
[4] B. Jungnitsch, T. Moroder, and O. Gühne, Phys. Rev. Lett. **106**, 190502 (2011).
[5] J. Gruca, W. Laskowski, M. Żukowski, N. Kiesel, W. Wieczorek, C. Schmid, and H. Weinfurter, Phys. Rev. A **82**, 012118 (2010).
[6] J.-D. Bancal, N. Gisin, Y.-C. Liang, and S. Pironio, Phys. Rev. Lett. **106**, 250404 (2011).
[7] H.M. Wiseman, S.J. Jones and A.C. Doherty, Phys. Rev. Lett. **98**, 140402 (2007);
[8] N. Brunner, D. Cavalcanti, S. Pironio, V. Scarani and S. Wehner, Rev. Mod. Phys. **86**, 419 (2014).
[9] J. F. Sturm, Opt. Methods and Software, **11-12**, 625 (1999). M. Grant and S. Boyd. CVX: Matlab software for disciplined convex programming, version 2.1. http://cvxr.com/cvx, March 2014.
[10] M. Horodecki, P. Horodecki, R. Horodecki, Separability of mixed states: necessary and sufficient conditions, Phys. Lett. A **223**, 1 (1996).
[11] A. C. Doherty, P. A. Parrilo, and F. M. Spedalieri, Phys. Rev. Lett. **88**, 187904 (2002); Phys. Rev. A **69**, 022308 (2004).
[12] A. C. Doherty, P. A. Parrilo, and F. M. Spedalieri, Phys. Rev. A **71**, 032333 (2005).
[13] M.F. Pusey, Phys. Rev. A **88**, 032313 (2013).
[14] M. Navascus, G. de la Torre, and T. Vértesi, Phys. Rev. X 4, 011011 (2014).
[15] M. Navascués, S. Pironio, and A. Acín, Phys. Rev. Lett. **98**, 10401 (2007); New J. Phys. **10**, 73013 (2008).
[16] O. Gühne and G. Tóth, Phys. Rep. **474**, 1 (2009).
